# The Asylums of Italy, France, and Germany

**Published:** 1859-10-01

**Authors:** J. T. Arlidge


### Art. V.—THE ASYLUMS OF ITALY, FRANCE, AND GERMANY.

BY J. T. ARLIDGE, A.B., M.B., L.R.C.P. (LOND.)

(*Continued from Vol. XI., p. 626.*)

### LYONS.

This, the second city in France, so well known throughout the world for its silk manufacture, and celebrated for ages as an emporium of commerce, possesses no institution for the insane which can be considered worthy of its civic importance and renown. Its past and present wealth is indicated by its public buildings and the extent of its quays; and its religion is symbolized by the gaudily decorated church crowning the hill of Fourvière, and itself surmounted by the gigantic gilded statue of the Virgin, the special protectress of the city, and the adored deliverer of its inhabitants from the plague of cholera, and of whose honour, glory, and miraculous virtues hundreds and thousands of votive offerings of wax models, tablets, and pictures, bear seeming ample witness to satisfy the most sceptical visitors of the church.

Near to this miracle-working edifice, on the brow of the same hill, overhanging the Saone, from whence a magnificent view of the great manufacturing mart is obtained, is a large, irregular, and sufficiently gloomy building known as the Hospice de l'Antiquaille, of which the lunatic asylum constitutes a considerable section. The buildings composing the Hospice are of very various dates; the foundations of the older portion, which was formerly a convent, having been constructed by the Romans at that early period when Lyons was the capital of Roman Gaul, and often the temporary residence of the Cæsars. However interesting these ancient foundations may be to the antiquary, and the comparatively modern conventual superstructure to the admirers of monastic life, they present no claim to the psychological physician, seeking information and suggestions for the improvement of asylum construction and arrangements.

The whole edifice, as before remarked, is not devoted to the purposes of an asylum for the insane, but serves besides as an infirmary for venereal and cutaneous disease, and for the old and vagrant population of the district, as in the case of the Hospice at Montpellier, described in my last paper. Like the latter, too, it is under one general administration, and the director, a non-medical official, superintends the whole institution, and is in all matters the paramount authority. The kitchen, laundry, and other domestic offices, the dietary, the economical details, and the means of warming and ventilating, are common to the whole establishment.

The insane population is lodged in some portions of the old conventual buildings, and in several wings or semi-detached buildings of later construction, among which a semicircular edifice has the especial merit of having been built for its present purpose.

It was not until about 1815 that the insane of the department of the Rhone, of which Lyons is the capital, were transferred to their present quarters; and however ill-suited and defective these may now be considered, they must have seemed to the poor creatures first introduced into them a real Elysium, compared with the prison-house and the horrible dens from whence they came. "When I first visited the insane (writes Esquirol) at Lyons, in 1809, they were received into the Hôtel Dieu and at the Hospice La Charité. At the latter institution they dwelt in the vaults beneath; at the Hôtel Dieu they were imprisoned in the thirty-eight rooms of a three-storied building, forming three sides of a narrow, irregular, and excessively damp court, in the centre of which was a well. The insane were never allowed outside their cells, at the windows of which they might be seen standing with their faces pressed against the bars. Those it might be wished to subject to the 'bath of surprise,' were conducted through the underground vaults to the Rhone." What became of them when conducted thus far, Esquirol does not state; whether their surprise ended in the plunge within the narrow bounds of a bath-house, or whether they were let contend against the stream, and struggle for a life not worth, one might almost say, the conservation, under the ever-enduring miseries it had to subsist in.

To return from this digression. The prevailing arrangement in the present asylum is that of the day and work-rooms on the ground floor, and the dormitories on the upper floors, with the exception of the dormitories for paralytics and feeble, demented cases and the infirmary, which are on the ground level. There is also a row of single rooms on the ground floor of a detached wing on the male, and in the section for the refractory on the female side.

The divisions for the two sexes are closely contiguous; that for the females is the superior of the two, and to it belongs the modern semicircular wing, built of late years, which has the most cheerful aspect of all the buildings appropriated to the insane. It has a corridor of communication, lighted from the court by windows sufficiently numerous, but closely guarded by outside venetian shutters, which replace the iron bars placed everywhere else to defend the windows externally, and protected by a net-work of wire on the inside.

On the female side are two workrooms on the first floor, besides the day rooms on the ground floor; these are the most comfortable and cheerful apartments in the establishment, and are occupied by those of a grade of society above the indigent who are found elsewhere. On the ground floor of this side there is also a folding-room, where some of the inmates are employed in folding and arranging the clothes. Another apartment on each side on the ground floor, is set apart for patients under restraint, who we regret to add are numerous. The rooms on the ground floor, particularly in the female division, have windows along only one side, an arrangement necessarily adverse to proper ventilation. The sitting rooms throughout the establishment were indifferently furnished; in the refractory departments with fixed clumsy benches and tables, and in the rooms of the quieter and better sort there was little other difference, except the presence of some chairs; the walls bare, without ornament or picture, save perhaps a crucifix or a Madonna, with artificial flowers, whilst the otherwise possible, extensive, and fine view from the windows was cut off by adjoining buildings or high walls. The dormitories are generally large and lofty, and like all other rooms have tiled floors, and are heated by hot air admitted through gratings placed at intervals along the skirting. The usual number of beds in a dormitory varies, according to its size, from twenty to forty. The bedsteads are very closely placed, leaving a clear space between them of not more than eighteen inches, an amount of crowding condemned by the visiting physicians. The bedsteads are of iron; and although the bottom is less than eighteen inches from the floor, yet the surface of the bed when put in order is above three feet from it, by the interposition of a very thick bag of straw or of shavings to serve as a palliasse, and of a thick wool mattress upon that. Each bed is supplied with an upper and an under blanket, sheets, and a blue cotton or linen coverlet, where the patient is clean; but where this is not the case, there is no under blanket, but the lower sheet is stretched immediately upon the straw which takes the place of the usual bed and palliasse, and is loosely contained in a zinc trough the size of the bedstead, into which it is fitted. The bottom of the zinc trough or box is so sloped as to conduct the urine which permeates the straw to a channel by which it escapes in a vessel beneath the bed. The straw, when wetted, is renewed every morning. This sort of trough and loose straw bed is used for paralytics; and it was stated to me that bed-sores were of very rare occurrence. The bedsteads used in the single rooms on the ground floor, appropriated to noisy and refractory patients, were exceptional in being made of wood. They followed the original trough or crib fashion, were very heavy and clumsy, securely fastened to the floor and wall, and duly furnished with rings for the paraphernalia of mechanical restraint, straps and bands.

The infirmaries differ in no respect from the dormitories, except in having a central stove, and, what must not be forgotten, a little cistern of water, with two taps and a sort of basin beneath, affording opportunity to wash the hands. As usual, the accessories, soap and towels, did not appear. I found the infirmaries, that on the female side more especially, close and hot. Provision is made in these apartments for the casually sick, for the paralytic, the aged, the feeble demented, and for some cases in which detention in bed is made to serve as a means of restraint.

Every dormitory and infirmary has a lantern suspended at the centre; but this imperfect means of illumination can only serve to make darkness visible. Two attendants in each division are on watch during the night, and perambulate the several apartments, and are especially charged to attend to those whose habits are bad, and to get them up once or more as occasion requires. The attendant also of each dormitory sleeps in a small room partitioned off from it, and is supposed to exercise a watch over its inmates through a small window which overlooks the room. The bath-house is a detached and indifferently constructed building, ill suited to its purpose. The baths themselves are of copper, short and narrow, but sufficiently deep. To every one is appended a clumsy wooden lid, which is used on every occasion, and the patient locked in under it by strong iron fastenings. Some short douche pipes were fitted over a few baths, but the douche was rarely used, and then not medically but as a means of repression and punishment. No shower-bath of the English model existed; but the effects of one were obtained at will by screwing a "rose" upon the end of a douche pipe. Nevertheless, here at Lyons, as at other places on the Continent, shower-baths were not so highly appreciated, and much more rarely used, than in England. They were held to be most beneficial in cases of melancholia. There were no water-closets within the building; these accessory structures were all outside, and may be briefly described as peculiarly unsavoury and French.

The airing courts were confined and small, surrounded by buildings or by high walls, dreary, neglected, and melancholy; their surface mostly uneven, and varied by the presence of holes, and when wet, with mud; in all respects calculated to render a monotonous, painful life more dreary and wretched.

It has been incidentally noticed that the dormitories are warmed by hot air entering by gratings placed along the skirting. In some of the sitting rooms the hot-air pipes pass along the centre, covered by iron plates; but besides these pipes the sitting rooms generally possess a central stove, surrounded and defended by a strong iron guard some five feet in height. The plan of heating by hot air is common throughout the Hospice.

The Hospice de l'Antiquaille is essentially an institution for the indigent, although the friends of some patients who have the means—a matter always determinable in France by the activity of the police, seconded by that of the Government—contribute to their support. It receives every variety and complication of insanity and idiocy, and is under the control of the central bureau for the administration of all the hospitals and hospices in the department. This Board makes the principal appointments, receives the reports of its officers, and sanctions all the details and all propositions affecting the working of the establishment. Under them is the non-medical director of the Hospice, on whom the general management devolves. The two physicians are simply chargeable with the medical oversight of the inmates, and with their moral discipline, their classification, their employment, the special diet of the sick, &c. The chief physician (in 1855) was M. Arthaud, charged with the male division, and his adjoint, M Lacour, superintended the female side. They both visited daily, when necessary twice; and were attended in their visits by the "internes" and a head attendant. The dispensary and dispenser serve the whole institution.

The male attendants wear a uniform dress, with a plated badge bearing a number. The nurses belong to a particular order, the "Sœurs Hospitalliers," a division, we believe, of the Sisters of St. Vincent de Paul, or Sisters of Mercy, who are devoted especially to the care of the sick in hospitals. The same order acted as nurses in the great hospital of Lyons—the Hôtel Dieu, containing 1300 beds. It is unnecessary to describe their peculiar dress, their extraordinary exaggerated caps, inasmuch as every visitor to France must have encountered some members of this sisterhood.

The number of patients at the Antiquaille, in 1855, was about 700, of whom 312 were males, and nearly 400 females. Nearly one-tenth of the males were stated to be paralytics, and about the same proportion epileptic. The classification adopted was into quiet and convalescent epileptics; noisy, and noisy and dirty, paralytic and dirty; it was found however not always perfectly practicable to carry it out. Those who were particularly noisy and troublesome were consigned to the single rooms in the basement, where they passed the night and more or less of the day, whilst in the state of excitement. The remainder of the refractory spent the day in the room set apart for them on the ground floor, and in the enclosed airing court attached to it. At night they were transferred to a dormitory, unless too riotous; indeed, except the few special cases referred to, the whole population of the asylum slept in dormitories. With respect to suicidal cases, these were placed among those who were lively and watchful, and some of them in the infirmaries.

The day was passed variously, according to the condition of the patient and the system and means of the establishment. Occupation was encouraged, and with much success among the females ; but for the males it was found difficult of attainment to any satisfactory extent. The only ground attached immediately to the asylum does not exceed three acres ; of this a portion is laid out as a kitchen garden, and gives employment to a few of the men. But there is an auxiliary establishment, consisting of a farm, which lessens this serious disadvantage of the confined site of the asylum itself. It is situated some short distance from the town, and occupies in its cultivation about twenty of the male patients. There is a small house upon it where the detachment resides from Monday morning to Saturday afternoon, the Sunday being spent in the town institution with the other inmates. More than the number named would most cheerfully be sent by the physicians, but there is neither accommodation nor occupation for more. This auxiliary branch, *i.e.*, with respect to economic and administrative details, was stated to work well with the parent institution.

The male patients who had to pass their whole time in the crowded, ill-ventilated, dreary day-rooms and pent-up courts of the Antiquaille, were indeed greatly to be pitied. No amusements seemed to be provided for them ; I saw no cards, no, not even dominos, which any visitor to France would set down as essential to a Frenchman's happiness. A very select few were permitted to read the few no doubt equally select books and journals which the asylum furnished ; and a small number was seen engaged in sticking wire through the pattern cards used in weaving.

This history of insufficient day-rooms, of gloomy airing courts, of the absence of the means of amusement or of employment, will prepare the reader to hear of the application of much restraint. This indeed was very largely the case. Many were to be seen confined by camisoles ; many fixed in strong arm chairs by straps, and often with the superaddition of camisoles ; some wearing besides a stout leathern stock, about three inches wide, around the neck, fastened behind by a strap to the chair, or otherwise to a belt extending upwards from another one around the waist. Others handcuffed and imprisoned in chairs, with or without the freedom of their legs ; and others, lastly, with more or fewer of the previously mentioned means of restraint, having in addition their feet manacled. Mechanical coercion, moreover, was not restricted to day use, but prevailed also at night, some wearing camisoles, others having their limbs fastened by straps ; and in either case, often further attached to the bedstead by other bands or belts.

The chairs in which so many had to pass their miserable existence during more or less of the day, and in the case of some, we fear, day after day, had their bottoms grooved and perforated, so as to allow the escape of the excretions into a zinc tray fitted beneath. Equally in pursuance of the same principle of applying mechanical contrivance in lieu of the supervision, the attention, and sympathy of fellow-men, the clothes of those lost to decency in their habits were made in one piece, and a sufficient opening left to permit the passage of the excreta.

Restraint is resorted to for the refractory, for some of the epileptic, for the paralytic under excitement, for the suicidal, and at night for those who would leave their beds. One homicidal man, who had once killed another, was pointed out as having had his arms constantly confined for several years. Excluding the particular class of patients of dirty habits and of those rendered so by confinement in chairs, the generality of the inmates were sufficiently and tolerably clothed. Most of the men had a suit of brown cloth; yet the dress was not uniform. The male epileptics wore a woollen cap, with a thickly padded wide rim, to screen the head from injury in their falls.

With respect to diet; soup is the staple article, made with or without meat, with vegetables, eggs, &c. They begin with a basin of soup at seven A.M.; at eleven A.M. comes another allowance of it, made from meat, with the wreck of the meat in the shape of "bouilli," and at four P M. a meat dinner arrives, without the potage being forgotten. Wine was too expensive to be served out, except in that finely diluted form of "eau rougi," which demands a French stomach for its appreciation. A certain modicum of bread, a larger proportion than commonly enters into the dietary of this country, constituted an accessory portion of each meal. Owing to the very thorough soddening and cooking of the meat, knives were not wanted, and forks and spoons answered every purpose.

Medicinal agents were not in much request. To open the bowels if costive, and to confine them if too open; to prescribe ptisans if feverish; syrup of marsh-mallow or some equivalent bland substance if a cough existed; these and similar simple measures appeared to constitute the bulk of the medical treatment pursued. Strychnine had been tried in paralysis without appreciable results; opiates were sometimes given to induce sleep, but much less frequently than with us in England, and strong coffee had been advantageously administered in cases of melancholia.

To sum up our impressions of this the asylum of the important city of Lyons. As its name, Antiquaille, suggests, there is a heavy brooding antiquity about it, both as a material edifice and as a living institution. It is a rusty antiquity, incapable alike of service and of serviceable repair. It perpetuates within it a system which has well nigh died out with advancing civilization, and this it does in a degree which we are glad to add is rarely seen elsewhere. There is positively nothing to be said by way of apology for the building. It is badly situated in a suburb of the city; has a very inadequate quantity of land attached to it; is, with respect to its architectural arrangements, very defective, and in its moral government ill-regulated. Even its only section, the semicircular building named, erected for the special purpose of an asylum, was long ago condemned by Esquirol as fraught with many disadvantages, notwithstanding the superiority of its arrangements and appearance over the other portions.

The confined site, the want of land to cultivate, the neglect of means of diversion for the inmates, their overcrowding, the insufficiency of male attendants, and the preconceived opinions and frequent impracticability of the religious sisterhood, a corps of *employées* in many matters independent in action of the medical staff of the institution, are among the chief causes of the excessive resort to mechanical coercion, in the place of which, moreover, the medical men doubtless can see no substitute, deficient as they are of experience in the management of asylums, possessing all the accessories of site, structure, space, and deprived of that unfettered power and rule with which the position of superintendent is rightly armed.

The office of director here at Lyons, as at many of the French asylums, resembles that of the governor of some of our oldest English asylums, and constitutes an insuperable difficulty to the free and effective medical and moral management of the establishment. The divided authority was felt and deplored as an evil; so was also the appropriation of the same institution to such incongruous purposes as those of an asylum, a Lock hospital, and a refuge and reformatory.

So far as it goes, the formation of an auxiliary to the parent Institution as a farm, is a step in the right direction, and shows that the necessity of better provision for the employment of the insane is recognised; but let us hope that it will shortly enter into the minds of the wealthy and numerous inhabitants of the second city of France, that a new asylum, well placed in the neighbouring country, well built, and well regulated by a medical superintendent, is an urgent need, indifference to which will bring down discredit and reproach upon them, among the best informed physicians and philanthropists of their own as well as of foreign countries.

### ASYLUM OF ST. JEAN-DE-DIEU, NEAR LYONS.

Some three miles from Lyons, on the road to Marseilles, is a large establishment for the insane of the male sex, belonging to the *frères* of "St. Jean de Dieu," which presents a favourable contrast to the town asylum of Lyons, just described. The building appropriated to this institution was originally constructed for the residence of a private gentleman, but has been much enlarged and fitted by means of various alterations to the purposes of an asylum by its present possessors, who have held it above thirty years. It is one of five similar establishments in France belonging to this benevolent and useful fraternity. The space of ground upon which the asylum stands is in the valley of the Rhone, and has a gentle slope towards the river, but is separated from it by a strip of flat land, formerly very swampy, aguish, and unwholesome, but now well drained. About ten acres of land belong to the asylum; they are enclosed by a high wall, and laid out in airing-courts and gardens cultivated by the inmates. The building is placed nearly in the centre of this space. The ground plan of the building is that of an elongated hollow square, having two wings extended from it on each side; one in a line with the principal front, the other from about the centre of the side, and parallel to the preceding. The chapel, surmounted by a fine tower, occupies the centre of the front elevation, and is placed somewhat in advance of it. The whole main edifice is of two stories, exclusive of an attic story not occupied by patients, and is in the Italian style of architecture. The walls on the inner front surrounding the enclosed court, are supported on arches resting on strong stone pillars, and in this way a continuous covered corridor or arcade is formed around the four sides. The general disposition of the apartments is such that all the sitting or day-rooms are on the ground floor, and all the sleeping rooms, with few exceptions, on the first floor. Next to the small room occupied by the doorkeeper, at the common entrance into the building, to one side of the chapel, is a large room where patients and visitors are received. On entering the corridor from the entrance hall, we come to the dispensary, consisting of three rooms, and next to it the kitchen; beyond are other offices. There is a dining-room to each division. The refractory are located in the posterior wing, of only one story, projecting at right angles from the main structure. The sleeping accommodation consists almost entirely of dormitories; but there is one section composed of some twelve single rooms, arranged on each side a corridor about 8 feet wide, having a window at the extremity opposite to the entrance door. Each of these single rooms was about 10 feet by 16-18 ft., and, like the corridor, some 14 feet in height; comfortably furnished and in nice order. The iron bedstead was fitted with curtains, the bed consisted of flock, and had a palliasse beneath. As at the Antiquaille, the bed was much elevated above the floor. The bedding consisted of upper and under sheets, a blanket, and coverlet. Besides the bed each room had a chair, a small table and mirror, and a strip of carpet by the bedside ; a crucifix, and often the picture of a saint, were placed on the wall. The floor, as everywhere else throughout the building, was paved with small red, square or hexagonal tiles, kept well dusted and polished, and set firmly in cement. The window was of the usual French casement fashion, opening inwardly, and defended externally by iron bars. These comfortable and well-kept rooms are only made use of as bedrooms, their occupants passing the day in the common sitting-rooms set apart for patients of the first class; for this asylum is of a mixed character, receiving a first and a second class of paying patients, and a third class wholly indigent, paid for out of the departmental resources, or, in a few instances, supported gratuitously by the brethren. The rate of pension varies from two to five francs per day. The second-class boarders, with few exceptions, sleep in dormitories, and have the same sort of beds and bedding, save curtains, as the first class. These dormitories are of very large size, being as much as 80 feet in length (probably more) by 18 or 20 feet in width. The bedsteads were arranged along each side, about 3 feet apart, and left a central avenue quite 8 feet wide, extending the entire length of the room. The area of these spacious apartments was interrupted about the middle by an abutment from the wall on either side, making a partial division into two rooms. The windows were large and sufficiently numerous, but ranged only along one side, excepting indeed at the upper and lower end. In some of the dormitories the external bars did not appear. The religious sentiment of the proprietors exhibited itself in the presence of a crucifix in every room ; also usually of a painting of some saint above the door, or of a small statue, and in the case of the bed coverings, of an embroidered cross, with or without the cyphers J.H.S. upon the woollen or linen coverlets. During the night a lantern suspended from the ceiling struggled by its tiny light with the surrounding darkness. The third-class dormitories were similar in size, but partially subdivided by incomplete partitions. The bedsteads and bedding were much the same, only that the latter was coarser. All the bedsteads appeared fixed to the floor. The Infirmary was merely a large dormitory, partially subdivided into three compartments. One of these was devoted to paralytic and dirty cases, and was shut off from the next by a glazed screen, whilst the partition between the second and third room was an open screen. A small altar, where mass was daily said for the sick, was placed at a central point so as to be visible from each division. The beds were wider apart than in the common sleeping rooms, and the bedsteads made of wood, stained and polished. Attached to one compartment of the infirmary were two or three single rooms, adapted for particular cases of sickness. Where the habits of the patients were dirty in bed, it was the plan to use a trough wooden bedstead, filled with straw, with a sheet below as well as above the patient. In feeble and paralytic cases, a folded sheet, in addition, was placed under the hips. The urine escaped into the straw, and through that, by a hole in the bottom of the bedstead, into a vessel beneath. The bath-house contained two bathing-rooms, one for the pensioners, the other for the indigent. The baths were deep, but short and narrow, of copper tinned inside, and not cased in wood, and severally furnished with a lid or cover. The baths for the third class had no separation between them, but those for the boarders had one curtain extended between them, and another across the foot; they also had convex metal covers in place of the flat wooden ones supplied in the other bath room. A douche pipe was suspended over the head of each bath, but was not sufficiently long or high above it to furnish a jet of much force; consequently, when it was wished to administer a douche, the patient was transferred to a section of the bath-room partitioned off from the rest, and supplied with a douche pipe of considerable force, together with an ordinary bath in which the patient could as usual be fixed by the aid of the cover fastened over it.

The usual classification of patients into quiet and convalescent, refractory, epileptic, paralytic, and dirty demented cases, was carried out, and to each class was appropriated its special quarter and its airing court. Although some of the patients had access to the central enclosed court, it was only exceptionally intended for their use, and was rather the office court, being in part surrounded by the general offices of the establishment. Of the other courts set apart for the patients, all, with the exception of the one on each side situated between the two parallel lateral wings, afforded some view of the neighbouring country. Unfortunately, their surrounding walls restricted the view very much, and it is to be regretted that in the case of some of the courts at least, sunk fences had not been adopted.

The chapel is of considerable size, and well built and decorated. The ground floor was set apart for the use of the brethren, whilst the patients occupied the gallery of a transept on each side, large enough to hold about one hundred seated on benches placed one above the other. Their view is very much restricted by a screen along the edge of the gallery, which at the same time acts as a guard against accidents by falling or leaping down into the chapel below. This screen is ingeniously constructed of stout deal boards, about twelve feet high and seven inches wide, placed at such an angle that all may see the altar, but not the congregation in the opposite transept or in the body of the church. To make doubly sure against accident, and to prevent any attempt to clamber over this screen, a stout polished cylinder of wood is extended across the transept above the screen, and so fitted as to revolve on being laid hold of, and to elude the grasp of the aspirant climber.

The whole land, as before remarked, was under careful cultivation. Although none of the best, being very sandy and loose, the portion occupied as garden ground was in good order. Besides the purely ornamental portions, there was one section set apart as a botanical garden. The airing courts were not satisfactory, being left bare except of a few trees. The vegetable garden was extensive, and a small portion of the land was cultivated as a farm. The cow-house, dairy, barn, &c., formed a detached building. All the work was done by the patients under the supervision of the brethren, who take them out daily in parties, the several classes being kept separate. Although gardening and husbandry supply the largest measure of employment, yet other occupations are not neglected. For instance, all the bread is made in the establishment, and shoemakers, tailors, and other workmen are enlisted for service in the workshops under the superintendence of mechanics hired from without. Carpenters are not much patronised because of the sharp tools necessary in their work. A certain number of patients are also employed in aiding the brethren in household duties, for everything affecting the management and order of the house devolves upon them, excepting always the supervision of the mechanical trades.

One great and sad want of this asylum is that of good water. They have to depend chiefly upon rain-water collected in a tank; for the Rhone water, obtained by sinking shallow wells, is not fit for use: an artesian well would probably remove this evil. The diet is very good, a portion of meat being allowed twice a day; the third meal is of soup without meat. There is also a liberal allowance of bread and vegetables: the physician considered the fare too liberal. Knives and forks were allowed to many; to the rest only spoons and forks. Amusements are provided,—at least for the pensioners, who have two billiard-tables, are allowed a few papers and books, and are permitted to gratify their taste for music and drawing. It is to be regretted, however, that the amusement of the indigent is not likewise attended to.

No general system of warming and ventilation was in operation. Every sitting-room had a central stove, surrounded by a guard, but, excepting the infirmary, no sleeping-room had any such provision.

All the brethren wear a similar monastic robe of coarse black cloth, with a hood, which serves as a covering for the otherwise unprotected head in bad weather. The patients have no uniform clothing;

The medical staff consists of a chief physician and an "adjoint;" one visits daily at eight in the morning, the other at four in the afternoon. Accommodation is provided in a small house at one corner of the asylum grounds, alongside the public road, for the physician; he, however, preferred to live in Lyons. The medical officers are paid servants of the *frères*, who exercise general control over the whole establishment, restricting the physicians pretty closely to the purely medical supervision. For although the classification and the employment of the patients and the imposition of restraint are assigned to the physicians as peculiarly their duty, yet the brethren take these matters pretty frequently in their own hands, chiefly, it must be admitted, during the absence of the medical men.

The *frères* appear to make very efficient and kind attendants on the insane. It is to this office, indeed, they specially devote their lives; they are a voluntary order of hospital attendants, and the performance of their duties is the subject of vows and of religious feeling. Nothing apparently could be more desirable than the enlisting of men as attendants on the insane, whose duties in the office would be their only coveted employment; whose end and aim would be their rigid and honest performance under an abiding sense of religious responsibility, coupled with a covenanted obedience to the supervision and control of one of their own number. But as there is no perfection in other human institutions, so this has its errors and evils. The community of nursing brethren at once opens the door to sundry failings and imperfections when it constitutes itself a body in authority, the ruler of an institution, and the master of others, and when questions of profit and loss have to enter into its calculations. The double part of attendant and master cannot be played successfully; and those who naturally should hold the reins of office, supervise the medical and moral treatment of the sick, dispose and arrange the institution in all its details for the furtherance of its objects as a medical and curative engine, are deposed from their proper position to be the dependants and paid *employés* of the corps of attendants.

The hindrances to the thoroughly efficient working of an asylum where the medical man is a sort of excrescence, and deprived of his independence of opinion and action by his subordinate position, need not be insisted upon at large. Suffice it to suggest the difficulties he must have to contend against when confronted by imperfect knowledge of asylum management, and of asylum wants, by the ignorance of medicine, and by unavoidable prejudices on the part of his employers. Or, again, how can a physician in such a position safely propose improvements or reforms which involve expense, even where profit is professed as only a secondary matter subservient to the extension of benevolent objects?

But to let these objections pass, others arise from other considerations. Religious motives and religious vows are in themselves fallible, and ever prone to degenerate into religious prejudice, pride, and bigotry; and thereby to counteract the best matured schemes and resolves. Members of religious orders, like soldiers, acquire an *esprit de corps:* they become partisans for the accepted predilections and motives of their fraternity, and devoted to its success. In this way erroneous opinions and prejudices gain firm possession of every member, and resist the attacks of any reformer, particularly when he is one bound by relation and position to obey. Again, if on this point in question we further take into consideration the connexion of these religious orders with the Roman Catholic Church, our argument against them in the capacity of administrators will be much strengthened. The interests of that Church must always be promoted, its jealousies always respected. Again, the usefulness of nursing fraternities is much diminished by the character of some of their members, and by the circumstances under which they have entered on their vows. Experience has shown that many persons unfitted in a moral and mental—and we may add in a physical—capacity, join themselves to these societies from various motives: some because they find themselves inapt for any ordinary remunerative occupation; others from a craving for seclusion and asceticism; others for a living, and to escape hard work as far as possible; others under religious excitement; and others from disappointment in business, in love, or other matters. Moreover, these religious societies require no previous education or mental enlightenment on the part of their novitiates; and hence the presence of a number of inefficient, perverse, ill-tempered, and obstinate folk in their community, and of not a few from the lower classes, who can neither read nor write, though sufficiently proficient in bigotry, prejudice, and superstition.

Lastly, to enumerate a few more evils of the system in question, the religious devotions, meditations, and fasts imposed by the Church and the religious order necessarily interfere with the management of an asylum; whilst the subjection of each member to the will of the superior in the house, and sometimes to one at a distant establishment, who can remove at will, and transfer him to another sphere of duty, takes away entirely that essential control the medical man should have over his attendants, and may at any time derange the efficient management of an asylum by the removal of a useful servant; or, on the other hand, by the substitution or retention of an inefficient or bad one.

To return from this long digression, two or three notes on this Asylum of St. Jean de Dieu remain for notice. Mechanical restraint is resorted to as a necessity in not a few cases. It is considered useful and necessary in maniacal excitement and in suicidal subjects. The latter sleep at night in the infirmary, where one or two attendants are constantly on the watch. When the propensity is very strong, they are often kept in the same apartment during the day. The utility of temporary seclusion to calm excitement is little recognised or practised, and the expedient of a padded room to defend the maniacal patient from self-inflicted injury has not been resorted to. The douche was employed as a measure of repression and punishment for refractory cases, and baths generally were in little request as means of treatment. I could discover no facts in respect of the medical treatment pursued deserving notice. The physician, however, ventured on a medical hypothesis in explanation of the prevalence of general paralysis in France. It was that, although intemperance was very operative, yet the abuse of mercury, chiefly in the treatment of syphilis, was very much more productive of this sad malady. To substantiate this opinion, he remarked that large numbers of persons affected with syphilis, unwilling to place themselves in the hands of physicians, resorted to the pharmaciens and other unqualified practitioners, who knew no other practice than that of giving mercury freely to produce salivation. Although there can be no doubt of the evil effects of mercury, so rashly administered, yet we fear it would be difficult to substantiate the opinion that it is causative of general paralysis; for the analysis of cases in this country would show that mercurial salivation was a rare feature in their history.

The number of inmates in this asylum at the date of my visit was stated to be 500, of whom some 70 were paralytic or epileptic. This population was more than double of that in 1838, when it stood at 208; and, to meet the increase, the building had been progressively augmented, and was, in 1854, still in course of enlargement. It admits the insane from any part of France, or indeed from abroad; but it especially serves as the asylum for the indigent of the departments of the Loire, de la Drôme, and du Gard.

A little *brochure* I picked up in Paris upon the statistics of this asylum, in the years 1838, 1839, and 1840, by the physician, M. Carrier, furnishes the following details:—On the first of January, 1838, the total number of inmates was 208, 87 of whom were pensioners, and 121 paupers. Of the 208, 59 were classed under mania—acute, chronic, intermittent, or complicated with epilepsy; 6 under monomania; 17 under melancholia; 4 as having hallucinations with delirium; 93 under dementia, including 8 epileptics and 2 paralytics; 4 under imbecility; and 25 as idiots. The admissions were, in 1838, 66; in 1839, 106; and in 1840, 89. They were most numerous in the spring quarter, in April, May, and June; next so in the summer months of July, August, and September; and least so in the last three months of the year; the difference, however, between the last-named period and the first quarter of the year in this particular being slight: with respect to the age on admission; 11 of the 261 cases were under 20; 67 between 20 and 30; 91 between 30 and 40; 17 between 50 and 60; 8 between 60 and 70; and one of each of the two next decennial periods. As to civil condition, 170 were unmarried, 86 married, and 5 widowed. The table of occupations shows that agricultural labourers were the most numerous—viz., 80; next to these, artisans, 61; then those without profession, 35; next, professional men, 22; and shopkeepers, 20; servants, 14. Hereditary tendency is noted in only 16 instances: moral causes are assigned in 74; excess in 45; organic changes in 50; external causes in 11; unknown, 64. The cures appear much influenced by the season: thus, in April, May, and June, 45 were discharged cured; 33 in the following 3 months; 30 in the last 3 months; and only 7 in the first quarter of the year. Looking to their civil condition, the married appear the most curable in proportion to the admissions. Among the deaths were 18 suffering from mania in its several forms; 2 from monomania; 5 from melancholia; 17 from simple dementia; 6 from dementia with epilepsy; 9 from dementia with paralysis; and 3 from senile dementia; also 1 imbecile and 2 idiots died. Death was attributed to cerebral disease in 21 instances; to chest diseases in 11; to abdominal in 23; and to various causes in 8. Reckoning that, in the course of three years, 469 cases were under treatment, and that 63 of them died, the death-rate is 1 in 15·22 for the year 1838; 1 in 17·83 for 1839; and 1 in 11·93 for 1840. Considered with reference to the number of admissions in each year, the proportion of deaths is as 1 in 26·5 for 1838; 1 in 26·5 for 1839; and 1 in 17·37 for 1840.

M. Carrier calculates the ratio of cures not on the whole population, but after the exclusion of justly-considered incurable cases—viz., demented patients in general; paralytics, epileptics, idiots, and imbeciles. Comparing the number remaining after this exclusion with that of the cures in the three years, he finds the proportion to amount to 1 in 4·57; or, taken year by year, 1 in 4·38 in 1838; 1 in 4·30 in 1839; and 1 in 4·71 in 1840.

Little general comment upon this retreat of the Brethren of St. Jean de Dieu is required. Great credit must be given them for its generally satisfactory condition, and it stands in highly favourable contrast with the asylum in Lyons, which it excels especially in its site and structure. One striking feature in the building is the vastness of the dormitories, which are the largest I met with among the many asylums I visited. They were well ventilated, very clean, and very neatly kept. Their elevation, not less than fifteen feet from floor to ceiling, greatly favoured their healthy ventilation, and afforded to the rooms a near approach to the standard cubic capacity laid down. Besides the two or more attendant brethren sleeping in each room, two others perambulated all the apartments, to watch and to attend to patients requiring their aid. Here, then, we see the dormitory system carried out on a very large scale; and if the physicians in medical charge of the asylum, and the brethren engaged in its actual working are to be believed, the results of this system are extremely satisfactory. The desire for privacy, in all probability, does not prevail so widely in France as in England; but, leaving this out of the question, the other objections brought forward against dormitories were distinctly stated not to be felt in practice, while, on the other hand, the easy and complete supervision, the influence of example, the withdrawal of patients from the solitude of single rooms, which to many give scope and occasion to delusions, fear, and frights, and the security against suicide, were urged as some of many advantages attending their use.

Although the promotion of employment, particularly of that out of doors, necessarily calls for commendation; yet the leaven of ancient prejudice is seen to remain in the objection of the brethren to the prosecution of trades requiring the use of edge-tools. Here is one instance in which the rule of such a brotherhood is, and is likely to continue for some time at least, an obstacle to the reception of those ideas of asylum management which are accepted by all medical men whose profession and position give them the benefit of observation and experience. The same comment applies also in the matter of mechanical coercion, here still extensively employed, and in almost all the forms which we have had to reprobate at the Antiquaille. The difficulty of persuading the monks to its abandonment will be so much the greater from their non-professional and general character, from their want of knowledge and experience, and from their interest in the *matériel* of the establishment, and in its preservation from damage by any means which may seem the most direct and efficacious.

We were pleased to find the recognition of so many excellent principles of moral treatment and management in M. Carrier's pamphlet. He insists on the advantages of regulated hours of employment, and of punctuality in the arrangements of the house, remarking that such constitute powerful means of regulating, and even of re-establishing, the healthy influence of the will, which, in all those bereft of reason, is always perverted or lost. "In every section (he adds) the meals, the walks, and the recreations are taken in common."

On the matter of treatment, M. Carrier very correctly remarks

"that to pretend to any exclusive method would be a fatal error. Experience, disengaged from all spirit of system, proves that the relief of the insane will invariably follow from a rational combination of moral and physical agencies, and that those cases are very exceptional where any one method of treatment can claim an absolute superiority. It is, moreover, the fact that phenomena which seem essentially of a moral nature are mostly complicated with evident derangement of some of the functions of organic life; so that, for instance, we have in one case agitation, sleeplessness, a large appetite, and constipation; in another, great physical apathy, drowsiness, the want of appetite, diarrhœa, &c. That this might naturally be expected, the enormous influence of the mind upon the health is sufficient to show . . . . To direct the senses of the insane to agreeable, and even, at times, to disturbing impressions—to adroitly and continuously divert their attention from the disordered conceptions which absorb their intellectual and moral powers—such, in a few words, is the system of the moral changes which, together, generally constitute the moral treatment of the insane."

There is, however, one method of treatment referred to by M. Carrier as occasionally practised in the asylum under notice—viz., that of *intimidation*, which it is enough to mention to secure its condemnation. There is yet another proceeding spoken of which we flattered ourselves had long since taken its place among the extinct barbarities of the past, but of which we are sorry to find a supporter in M. Carrier—viz., the "bath of surprise." This physician says: "I could cite a good number of cures effected almost instantaneously by this powerful means of treatment in cases of mania and of monomania." Notwithstanding this flattering report in its favour, no one, we feel assured, will now-a-days have the hardihood to revive this most reprehensible proceeding with the belief that he is to benefit his patients by it. Where fright cures one case of brain disorder, we may safely aver that it causes a hundred.

Examinations after death, M. Carrier tells us, "have almost constantly revealed the existence of lesions of the brain, or of its membranes. Yet there is little constancy of relation discoverable between the form of the delirium assumed and the seat and nature of the pathological changes. One fact I would point out as well deserving notice—viz., that insanity, the consequence of the exhibition of mercury, has, in all instances where death has followed, always been marked by a chronic form of hydrocephalus, without appreciable alteration either of the brain or of its membranes . . . . Pulmonary consumption has frequently been associated with melancholia, and a sort of adynamic dysentery has hastened the termination of numerous cases of dementia."

One word more with reference to M. Carrier's opinions. He is an advocate for the complete separation of the two sexes, by placing them in distinct asylums; for he is particularly apprehensive of unruly passions,—so often operative in the causation of insanity, being excited by the vicinity of the opposite sex. Some allowance must be made for this decisive opinion, seeing that the writer is, to a certain extent, the advocate of a particular asylum where females are rigorously excluded—a circumstance, by the way, we imagine due rather to the rules of the religious order who govern it than to medical or psychological considerations. Be this as it may, an *amour propre* for the institution he serves, and the want of experience in any asylum where not only are the males and females located under the same roof, but, under proper supervision, and at certain periods, brought together in the common enjoyment of recreations and amusements, will afford some apology for his adoption of the principle of complete isolation of the sexes.

At some little distance from this Asylum of St. Jean de Dieu, on the same road, but nearer to Lyons, is a small community of "Religieuses," of the order of St. Vincent de Paul, who devote themselves to the care of the insane, and had, in 1854, about thirty female patients in their house. The building, which was close to the high road, was, in the year named, in course of extensive enlargement, to receive an increased number of patients. Being so small an institution, and knowing that all such institutions belonging to religious orders can only be exceptionally visited, particularly when under female control, I made no attempt to inspect it. As it may, however, have by this time, according to the apparent law of asylum growth, have increased to the dimensions of a large establishment, it will be worth inquiring after by any psychological physician who may be staying for a few days in Lyons.

